# Transcriptome Kinetics Is Governed by a Genome-Wide Coupling of mRNA Production and Degradation: A Role for RNA Pol II

**DOI:** 10.1371/journal.pgen.1002273

**Published:** 2011-09-08

**Authors:** Ophir Shalem, Bella Groisman, Mordechai Choder, Orna Dahan, Yitzhak Pilpel

**Affiliations:** Department of Molecular Genetics, Weizmann Institute of Science, Rehovot, Israel; Stanford University School of Medicine, United States of America

## Abstract

Transcriptome dynamics is governed by two opposing processes, mRNA production and degradation. Recent studies found that changes in these processes are frequently coordinated and that the relationship between them shapes transcriptome kinetics. Specifically, when transcription changes are counter-acted with changes in mRNA stability, transient fast-relaxing transcriptome kinetics is observed. A possible molecular mechanism underlying such coordinated regulation might lay in two RNA polymerase (Pol II) subunits, Rpb4 and Rpb7, which are recruited to mRNAs during transcription and later affect their degradation in the cytoplasm. Here we used a yeast strain carrying a mutant Pol II which poorly recruits these subunits. We show that this mutant strain is impaired in its ability to modulate mRNA stability in response to stress. The normal negative coordinated regulation is lost in the mutant, resulting in abnormal transcriptome profiles both with respect to magnitude and kinetics of responses. These results reveal an important role for Pol II, in regulation of both mRNA synthesis and degradation, and also in coordinating between them. We propose a simple model for production-degradation coupling that accounts for our observations. The model shows how a simple manipulation of the rates of co-transcriptional mRNA imprinting by Pol II may govern genome-wide transcriptome kinetics in response to environmental changes.

## Author Summary

Organisms alter genes expression programs in response to changes in their environment. Such programs can specify fast induction, slow relaxation, oscillations, etc. Conceivably these kinetic outputs may depend on proper orchestration of the various phases of gene expression, including transcription, translation, and mRNA decay. In particular, in the transcriptomes of a broad range of species, fast mRNA “spikes” appear to result from surprisingly “pressing the gas and the brakes” together, i.e. by activating both transcription and degradation of same transcripts. A recently discovered molecular mechanism, in which subunits of RNA polymerase II (Pol II) associate to mRNAs during transcription and control their decay, could explain how such transcription-decay counter-action works. Yet, how such potential coupling responds to physiological conditions and how it shapes transcriptome kinetics remain unknown. Here we used a minimalist mutation in yeast RNA Pol II that is defective in the above mechanism in order to show that Pol II governs the ability of the cell to modulate mRNA decay in stress and, most importantly, that Pol II is essential for appropriate coupling between mRNA production and degradation. We further show that this transcription-decay coupling is responsible for shaping the transcriptome kinetic profiles under changing environmental conditions.

## Introduction

The dynamics of the transcriptome in response to environmental changes is chiefly governed by two opposing processes – RNA production, namely transcription, and RNA degradation. Despite this fact, most of the attention has been given to the study of transcription. Recently genome-wide techniques have been established that allow to measure separately the contribution of mRNA degradation [1]–[4] and transcription [5]–[8] to the balanced mRNA levels in the cell. Such studies revealed extensive regulation on both production and degradation rates. In particular, it became apparent that mRNA degradation is heavily regulated - genes that belong to the same complexes or gene modules, such as the ribosomal proteins or the proteasome, were shown to be co-degraded in several conditions and are considered to be part of the same decay regulon [2], [9], [10]. In addition, the decay rates of some genes across various growth conditions showed extensive variation, featuring stabilization in some conditions and de-stabilization in others [9], [11], [12].

Yet the emerging picture from many of these studies is that in addition to heavy regulation on both levels of production and degradation, there is often a correlation between the regulation of the two levels. In particular several studies have shown a “counter-action” mode of coupling between the two levels of control. In this mode of coupling genes that are induced at a given situation undergo, somewhat surprisingly, de-stabilization. The outcome of such type of coupling appears to be a fast transient change in mRNA abundance. This notion was demonstrated in the yeast *Saccharomyces cerevisiae* [9], [10] in *Saccharomyces pombe* [13] and in mammals [14]. Interestingly, counter-action is not the only mode of coupling of production and degradation of mRNAs. Genes that show a slow, and sustained dynamic change in mRNA levels in response to a certain stimulus typically display the opposite and more intuitive correlation by which changes in mRNA abundance and stability are in the same direction [5], [9]. The above results indicate that regulation of mRNA stability and its mode of coupling with transcription have a major contribution to the shape and dynamics of the transcriptome response to the environment [9], [13], [15], [16].

A major question is thus – is there a molecular mechanism in cells which ensures the coupling between transcription and mRNA decay. A potential mechanistic basis for such coupling can be suggested based on detailed biochemical analysis of individual genes in the yeast *S. cerevisiae*. It was recently shown that, in addition to their genome wide role in transcription [17], [18], two subunits of the RNA polymerase II (Pol II), Rpb4 and Rpb7, which belong to the holo-enzyme, not the core enzyme, can associate with the transcript during transcription and later chaperone it to the cytoplasm [19]–[22]. Crystal structure analysis of Pol II supports this notion since Rpb7 was shown to interact with the nascent transcript as it emerges from the core polymerase [23]. In addition, *in vitro* studies, using proteins extracted from human cells, demonstrated that hsRpb7p interacts with the transcript as it emerges from Pol II [24]. Additional support for the role of Rpb4 in post-transcription control came from the realization that it recruits to the mRNA 3′ processing and polyadenylation enzymes [25]. Chaperoning of the transcript to the cytoplasm by the two polymerase subunits may affect a diversity of post-transcriptional process including translation and mRNA degradation [22]. These two subunits of Pol II may thus implement a simple means of coupling between transcription and mRNA decay. To show that the cytoplasmic role of Rpb4/7 depends on its nuclear association with Pol II core subunits in the nucleus, Goler-Baron et al [19] used a mutant in a subunit of the core polymerase, Rpb6. In this mutant, a glutamine at position 100 in Rpb6 was replaced with an arginine. This mutation displayed reduced ability to recruit Rpb4/7 both to the core polymerase [26] and to mRNA transcripts [19], [22] which resulted in impaired production and degradation for a selected group of genes. The mutant thus seem to hold a key to understanding the coupling since it represents a minimalist perturbation that could de-couple genome-wide transcription from degradation, while maintaining intact Rpb4 and Rpb7 in the cell.

Here we used this minimalist perturbation to reveal a role of Pol II in affecting mRNA degradation and coordinating this process with transcription on a genome wide level. For that we compared gene expression, and decay rates in optimal growth conditions and in stress in the rpb6 mutant and a wild-type strain that is otherwise 100% identical to the mutant throughout the genome. We found that the rpb6 mutant is compromised in its ability to module mRNA decay rates, in addition to impaired transcription, in response to stress. As a result, while the wild-type features a counter-action coupling between production and degradation of mRNA, in the mutant this coupling is lost. This loss of negative correlation between changes in mRNA abundance to changes in stability in response to stress, results in an impaired temporal mRNA abundance response both in magnitude and kinetics. We thus conclude that in addition to its prime role in transcription, Pol II, also affects mRNA decay genome-wide, in a way that provides coupling of the two processes. This coupling appears to shape the kinetics of the transcriptome in response to stress. We propose a simple model which explains this coupling and its effect on the transcriptome.

## Results

### The experimental design

**Figure 1:**
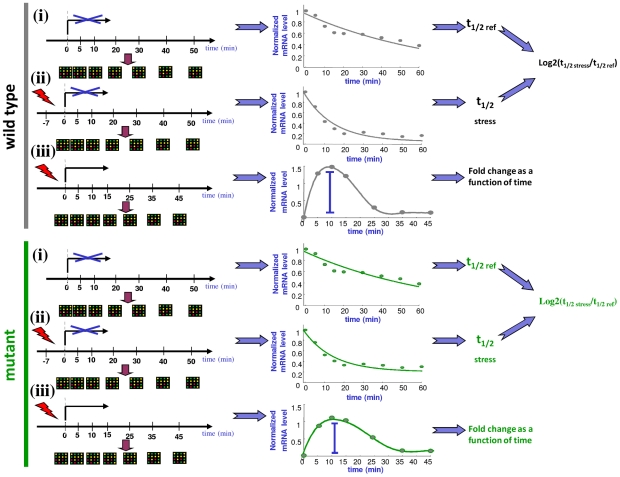
A schematic illustration of the experimental procedure. For each strain three types of experiments were conducted: (i) a reference decay experiment where decay kinetics was measured after transcription inhibition without applying additional stress. (ii) A stress followed by transcription inhibition to measure condition specific decay kinetics, and (iii) A conventional microarray experiment where mRNA abundance was measured following the perturbation.

To check whether Rpb4/7 has a role in the previously observed counter-action coupling of mRNA production and degradation in stress [9] we used the rpb6^Q100R^ mutant previously described [26] and compared it to its parental isogenic wild-type strain that is other-wise 100% identical throughout the genome. Pol II containing this mutant subunit has a reduced ability to recruit Rpb4/7 subunits to transcripts [19], [22]. The experimental setting is described schematically in Figure 1. Briefly, for both the wild type and rpb6^Q100R^ mutant strain we applied oxidative stress to two cultures, one for a conventional mRNA abundance profiling and one in which transcription was inhibited using 1,10-phenanthroline [1], 5–7 minutes after applying the stress, for mRNA decay measurement (see Materials and Methods). Following the addition of the drug mRNA levels were measured in five time points using the same microarray procedure as for the mRNA abundance profiling which did not involve transcription arrest. In addition we also measured for each strain mRNA stability without an oxidative stress, producing a reference decay profile for each gene. Decay profiles were fitted to an exponential decay model from which half-lives were calculated for each gene in each strain and condition (see Materials and Methods). The majority of the genes in the genome (>90%) showed a good fit to an exponential decay model (see Materials and Methods).

In another stress condition, the DNA-damaging drug MMS, which we have tuned before to invoke a qualitatively different transcriptome kinetics compared to the response to the current oxidative stress [9] we also compared the mutant and the wild-type though only in conventional mRNA profiling, without transcriptional arrest.

### Rpb6 mutant is defective in counter-acting changes in mRNA production and degradation in response to stress

**Figure 2:**
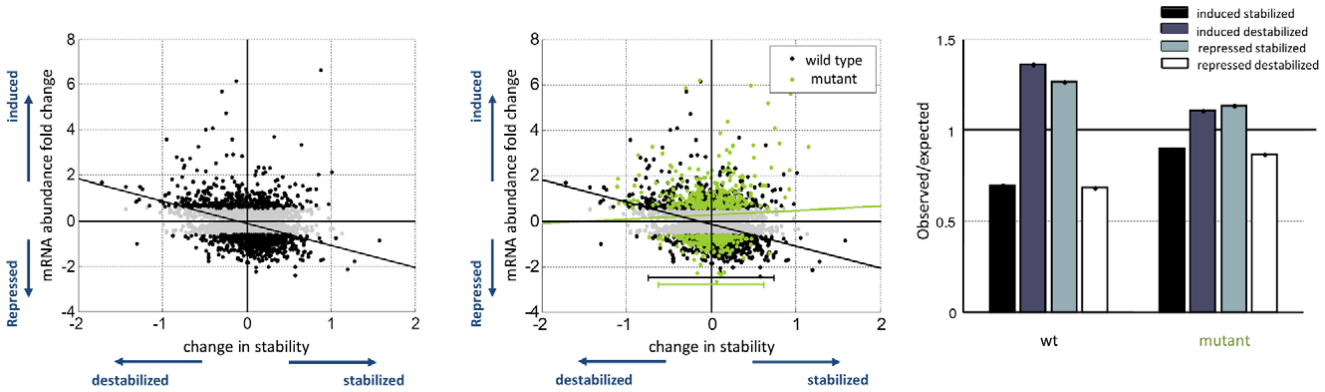
Reduced coupling in Rpb6 mutant strain. The change in mRNA stability relative to the reference state (
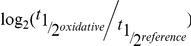
) is plotted against the maximal fold change, defined as the maximal change in mRNA abundance for each gene across the time course. (A) Shows the wild type strain where black dots marks genes which respond to the stress. A negative correlation bywhich induced genes are destabilized is illustrated by the plotted least square line (R = −0.23, −log_10_(p-value)>58). Fitted line y = −0.97x−0.12 with (−1.14, −0.7947) and (−0.1875, −0.05864) 95% confidence interval for each parameter. (B) The mutant measurements are plotted on top of the wild type. The negative correlation observed in the wild type, reflected by the black least straight line, is almost completely eliminated, displayed in the green least square line (R = −0.06, −log_10_(p-value)<6). Fitted line y = 0.1852x+0.27 with (−0.1723, 0.5426) and (0.1596, 0.3805) confidence interval for each parameter. Also the width of the distribution across the x-axis is slightly narrower for the mutant strain an indication for reduced ability to modulate mRNA stability. (C) Fold enrichment for the change in stability in induced and repressed genes. The number of observed stabilized and destabilized genes within both induced and repressed groups of genes divided by the expected number, assuming no correlation. Expected number is calculated as the percentage of stabilized/destabilized genes in the genome times the induced/repressed group size.

We computed for each gene in each strain the mRNA abundance maximal fold change in response to stress, a measure that can be affected both by changes in transcription and degradation. In parallel, to isolate response to stress at the degradation level, we characterize each gene in each strain by the ratio between its half-life in oxidative stress to its half-life in the reference condition. Figure 2A summarizes the data for the wild type strain: we observed a negative correlation between mRNA fold change and stability changes, i.e. induced genes show a tendency towards destabilization while repressed genes show a tendency to be stabilized in response to oxidative stress. This negative correlation is in agreement with our previous finding using slightly different experimental setup and a different genetic background [9]. In Figure 2B the same analysis is presented for the mutant on top of the wild type. This comparison reveals an almost complete elimination of the counter-action coupling that we observed in the wild type between mRNA abundance fold-change and stability change; this same result is presented in a more quantitative way in Figure 2C by calculating the observed vs. expected association between changes in production to changes in degradation assuming independence between these two measures (fold enrichment). In addition, the mutant shows a reduced capacity to change mRNA stability in response to stress, both extreme stabilization and extreme de-stabilization is not seen in the mutant (Figure 2B). Such a reduced capacity to modulate stability due to a mutation in Pol II is remarkable and by itself intriguingly suggests an effect of RNA Pol II on mRNA degradation.

These results are an indication that the counter-action mode of coupling may indeed require coordination between transcription and degradation and they further suggest that recruitment of Rpb4/7 to Pol II is important for the coupling mechanism.

### Rpb6 mutant displays an impaired mRNA abundance temporal response to stress

We suggested earlier that the counter-action mode of coupling between production and degradation may be responsible for the spiked and fast relaxing dynamics on both induced and repressed genes in response to oxidative stress [9]. Rpb6 mutant provides a good opportunity to examine whether lack of coupling results in a less spiked and slower relaxing temporal mRNA abundance profiles. Yet, since the mutant is defective both in mRNA synthesis and stability we expect a more complex change in expression profiles, i.e. both in the magnitude and kinetics of the response.

**Figure 3:**
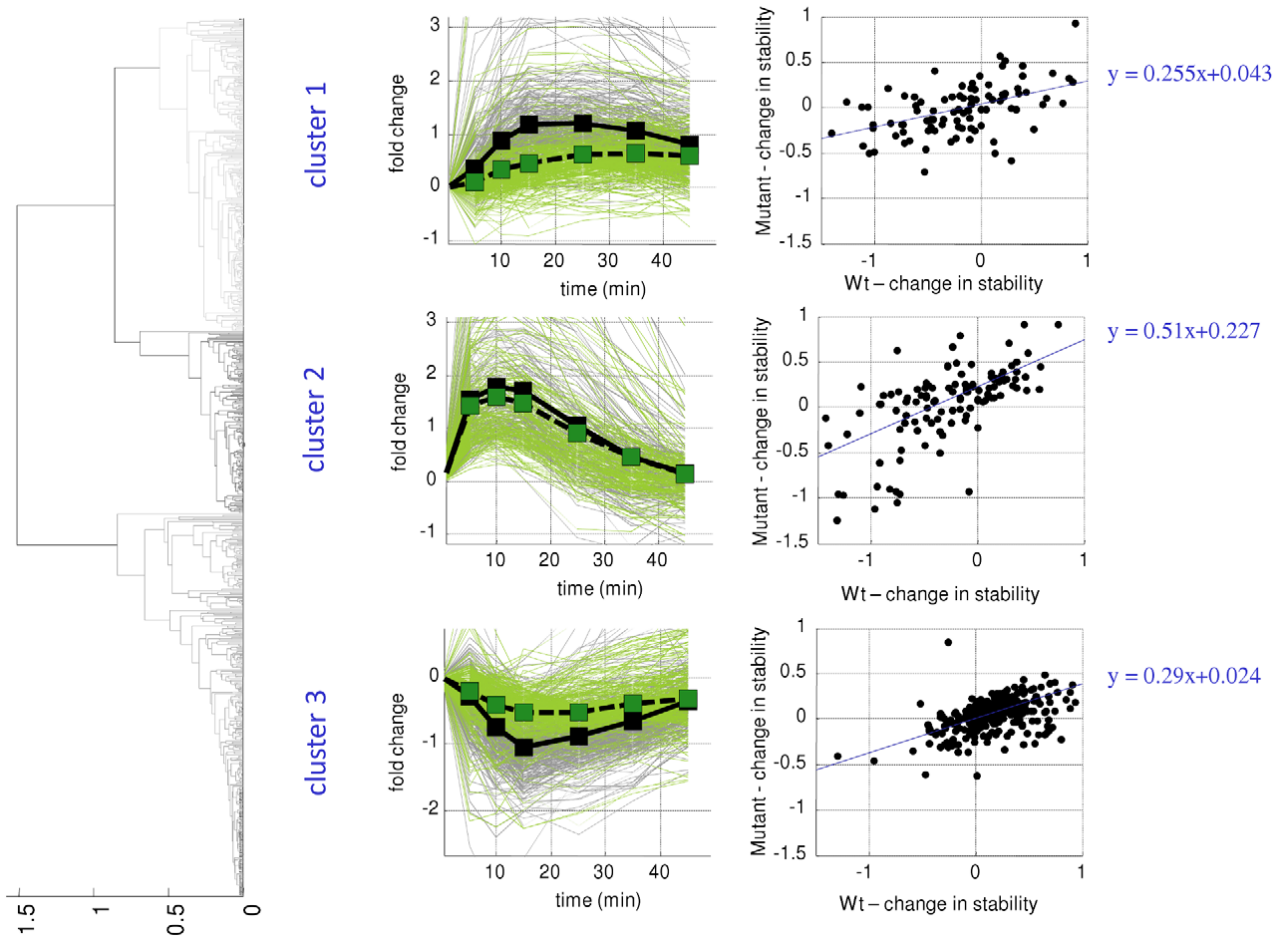
Impaired response of Rbp6 mutant strain. Hierarchical clustering of mRNA abundance temporal profiles. The three clusters marked in the dendrogram by different gray colors correspond to the three clusters in the middle and right panels. Middle panels show the fold change as a function of time for both strains, gray for wild type and green for mutant, with thick lines representing the mean of each strain. Left panels show the difference in stability in response to stress, calculated as the log2 ratio of the stress half-life by the reference half-life. The wild type value is plotted against the mutant with a blue least square line showing the general trend for each cluster. To show the slope difference between the two induced clusters fit parameters are also displayed for all clusters.

To show the effect of the mutation on the entire genome and on different groups of genes we clustered concatenated wild-type and mutant mRNA abundance profiles for the stress-responsive genes (see Materials and Methods). As shown from the dendrogram in Figure 3, most of the stress-responsive genes reside within clusters that show a difference between the wild type and mutant (1 and 3). As expected the difference is displayed both in the magnitude of the response and in its kinetics. Reassuringly, for the induced genes that show a difference between the wild-type and the mutant, the difference in the kinetics matches with the expectations given the reduced destabilization: we see a less transient response in the mutant, which might be the effect of delayed de-stabilization-dependent relaxation of the early response and longer response time. To emphasize the differences in kinetics we also plot the mRNA abundance profiles of cluster 1 normalized by the standard deviation for each gene (Figure S1) which reveals more clearly the shift toward a slower response for induced genes. For repressed genes too most genes show reduced repression and a slight shift in response kinetics towards a more sustained and less transient behavior. In the case of repression the difference is more apparent in the magnitude and less in the kinetics. A possible explanation might be that reduced repression is a result of the lower basal level for these genes, see below.

It is important to mention, that actually the fastest responding and relaxing induced genes show the least degree of change due to the mutation (cluster 2 in Figure 3). This, at- first- surprising, observation could be explained by the nature of the mutation which only reduces, but does not eliminate, the recruitment of Rpb4/7 to transcripts. According to this possibility the fastest responding genes have the highest ability to recruit Rpb4/7, and hence is their ability to still recruit these two subunits in the mutant too. Clearly an alternative explanation might be that for these genes the transient behavior is independent of Rpb4/7 (see also Discussion). We performed a GO enrichment analysis to characterize the functional association of the genes in each of the clusters shown in Figure 3 (Table S1). Reassuringly we find a typical response to stress by which stress-related genes are induced while growth-related genes are repressed.

In order to show that reduced ability to modulate mRNA decay in the mutant is indeed associated with, and perhaps even causative of, the observed shift in kinetics, we compared for each cluster the change in stability in response to stress between the two strains (right panels of Figure 3). A slope smaller than unity, in the linear fit, represent an impaired ability to modulate stability in the mutant compared to the wild-type. Reassuringly, comparison of the two induced clusters shows a significantly reduced slope for the cluster that shows the difference in mRNA abundance temporal profiles between the strains. This result shows how the reduced ability of the mutant to destabilize induced genes might in part be responsible for the shift of these genes from spiked to slower response and relaxation kinetics. A reduced change in mRNA stability is also observed in the cluster that shows similar mRNA abundance profiles between the wild-type and the mutant. In this case, in addition to the above explanations, compromised transcription might compensate for the change in stability.

We also experimented with an additional stress, the DNA damaging agent MMS, which in our earlier work showed sustained and long enduring expression profiles [9]. In the present experiment the mutant and the wild-type showed very similar expression profile, with only a small reduction in the magnitude of the response in the mutant (not shown, and data deposited publicly, see Materials and Methods). This observation could indicate that the long enduring response displayed in this condition is independent of a coupling mechanism between production and degradation (or at least that it is not dependent on Rpb4/7 in that condition). We thus decided not to carry out decay experiments in the mutant under this condition.

### Reduced recruitment of Rpb4/7 in the mutant results in a reduction in both production and degradation in basal conditions

**Figure 4:**
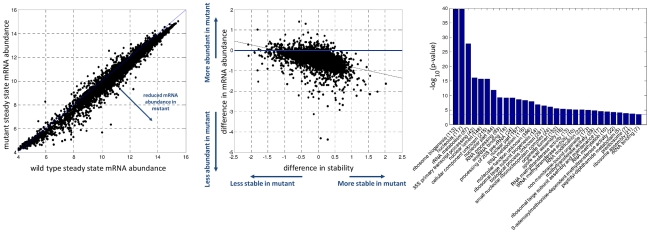
Wild type and mutant differences in basal conditions. (A) correlation of steady state mRNA abundance measurements between the wild type and mutant strain. A global reduction in mRNA levels is observed in the mutant. Data is plotted in log2 scale. (B) Basal difference in stability (log 2 ratio of the reference half-lives of the mutant divided by the wild type) is plotted against the difference in mRNA abundance (log 2 ratio of basal mRNA abundance in the WT and mutant). Most changing genes show both a reduction and stabilization in the mutant strain. The black least square line shows the negative correlation between these two parameters. Fitted line y = −0.3986x−0.4557 with (−0.4183, −0.3789) and (−0.4636, −0.4477) 95% confidence intervals for each parameter. Blue line represents no change in mRNA abundance. (C) Results of a gene ontology enrichment analysis for the genes that show the largest reduction in mRNA abundance in the mutant strain.

A difference between the two strains can also be observed at the reference un-stressed conditions. To study the basal difference in mRNA abundance between the two strains in optimal conditions the mRNA abundance levels at time point zero (before addition of the stress) in the two strains where normalized using spiked-in RNA internal standard. A unique normalization procedure is required here because standard microarray normalization methods assume a constant global distribution of intensity values between samples preserving ranking differences between genes but eliminating global differences (see Materials and Methods). While gene expression levels strongly correlate between the two stains, we observe an overall reduction in mRNA levels in the mutant vs. the wild-type, as expected from a mutant in Pol II (Figure 4A). This observation extends previous reports of reduced production in this strain [27], showing this effect on a genome wide level.

We next asked how the difference in steady state mRNA abundance relates to differences in stability by plotting the two parameters against each other. Figure 4B shows how genes with reduced mRNA abundance in the mutant actually become more stable, opposite of a potentially intuitive relationship but in line with the combined effect of Rpb4/7 on both production and degradation [19], [21]. The large reduction in mRNA abundance, despite the elevation in stability, indicates that a larger decrease in production is taking place - otherwise the net decrease in mRNA levels would not be observed. Genes displaying increased destabilization due to the mutation are expected to show a reduction in mRNA abundance which is not observed in this plot. The reason resides in the normalization procedure which artificially centers the differences in stability around zero, thus correcting a probable shift of the values towards less degradation in the mutant (see Materials and Methods).

We thus conclude from this plot that the genes which are most affected by the mutation at optimal conditions are the genes which show a decrease in mRNA abundance coupled with stabilization. We find that those genes are highly enriched with ribosome biogenesis GO functionalities and other specific functional groups (Figure 4C), in line with previous observations relating stability of mRNAs encoding ribosome biogenesis factors with rpb4/7 functions [20]. This observation goes along with a slower growth rate for this mutant of about 40% relative to the wild-type.

## Discussion

Previous studies of Rbp4/7 proposed for the first time a biochemical mechanism in which Pol II affects mRNA degradation by imprinting mRNA transcripts with two of the polymerase subunits that escort mRNAs to the cytoplasm [28]. In this work we show that the process of coupling transcription and mRNA decay is a genome-wide, regulated process that depends on the physiology of the cell and on the environment. Further, we show that this mechanism shapes the temporal kinetic response of mRNA abundance to changing external conditions.

**Figure 5:**
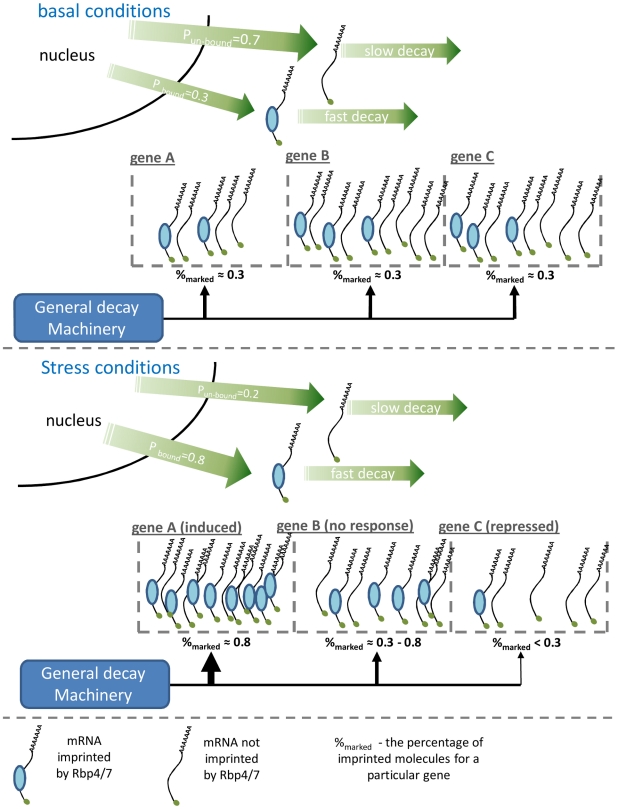
A schematic illustration of the suggested model explaining the genome-wide effect of Rpb4/7. The figure shows how an increase in the association probability following stress causes a global redistribution of the general decay machinery resulting in genome wide coupling of changes in mRNA abundance and stability. Theoretical probability for a transcript to be either imprinted or non-imprinted by Rpb4/7 during transcription in basal conditions (A) and for stress (B). A more detailed explanation is given in the main text.

To explain how Rpb4/7 has a genome-wide effect on mRNA stability, and in opposite directions for induced and repressed genes, we propose a simple molecular model, described schematically in Figure 5. Previous molecular work shows that an mRNA molecule can be exported into the cytoplasm either associated (imprinted) or not associated with Rpb4/7 and that imprinting will result in an increased probability to be degraded [19], [21]. Let us assume that the general factors responsible for mRNA decay in the cytoplasm are in limited amount and are distributed across the transcripts according to each transcript's ability to recruit these factors. Because Rpb4/7 imprinted mRNAs have increased ability to recruit general decay machinery, the distribution of the general decay machinery across different genes will be in proportion to the fraction of Rpb4/7-imprinted mRNA molecule copies for each gene. Assume that in basal conditions we have some probability (e.g. 0.3) for an mRNA to be exported into the cytoplasm imprinted by Rpb4/7. This will give rise to a similar percentage of Rpb4/7 imprinted mRNA molecules per gene in the cytoplasm which, assuming no other effects, will result in a uniform distribution of decay resources over all genes (Figure 5A).

Now let us assume that in stress the probability of imprinting by Rpb4/7 is increased (e.g. p = 0.8) per individual transcript molecule. This can be easily achieved by increasing the concentration of any cofactor contributing to Rpb4/7 imprinting. Following this change, genes which are induced due to the stress will quickly increase the percentage of imprinted mRNA molecules, thus gaining a higher affinity to general decay machinery which will result in faster decay for these genes (gene A in Figure 5B). Such genes would usually be also present in lower copies in basal conditions as depicted in Figure 5A. Genes which are not induced at the transcription level in response to the stress, yet are still transcribed at lower rates, will slowly increase the percentage of imprinted mRNA molecules resulting in intermediate decay (gene B). Repressed genes, for whose transcription is decreased, will keep the basal percentage of imprinted molecules and will even experience a decrease in this percentage due to the faster decay of imprinted molecules, which will result in general slow decay for these genes and a transient decrease in numbers. Thus, by only increasing the probability of Rpb4/7 imprinted export we get redistribution of the general decay machinery in favor of induced genes. Increasing it during a response to stress will result in coupling between mRNA production and degradation and a genome wide fast transient adaptive response. In support of this model, previous works have indeed shown increased Rpb4/7 mediated export in stressful conditions [29], [30].

While a lot is known about the control of each stage of gene expression in isolation, recent accumulating knowledge suggests that many of the stages are coupled [31]–[33]. Using the rpb6 mutant it was shown that reduced ability to recruit rpb4/7 to the core polymerase results in impaired production and decay for selected genes [19]–[21]. Here we show that this single amino acid substitution in Pol II, which likely decouples transcription from mRNA decay, has a genome wide effect on gene expression both under optimal conditions and in stress. This mutant is defective both in mRNA synthesis and decay and has a reduced ability to modulate mRNA decay under stress conditions. The impaired ability to affect production and degradation is reflected in the mRNA abundance temporal response to stress by which most responsive genes, induced and repressed, show a difference in the magnitude and the kinetics of the response – towards lower fold change difference and slower relaxing kinetics. While a genome-wide decrease in production due to a mutation in Pol II is expected, the large effect that this mutation has also on mRNA stability and the loss of correlation between the changes in stability and production is intriguing. This, together with the accumulating molecular evidence [19]–[21], indicates that Rpb4/7 not only affects different regulation levels, as production and degradation, but also serves to coordinate between these levels as has been recently proposed [22]. We note though that we cannot exclude an alternative in which due to a defect of the mutant in transcription the mutant does not transcribe machinery that is required for normal decay of mRNAs.

It should be noted that while most responding genes were affected by the mutation, some did not, especially the most fast-relaxing transient genes, genes that also show a strong counter-action between mRNA abundance levels and decay (Figure 3). This could indicate that these genes are not clients of the *RPB4* and *RPB7* chaperoning mechanism and may owe their strong counter action and fast response to another mechanism. Alternatively the lack of sensitivity of the most rapidly responding genes to the mutation could simply indicate that the Q100R mutation did not abolish the normal dynamics of these genes, presumably because these genes represent the strongest-affinity clients of this coupling machinery, this possibility should be examined experimentally for individual genes.

Tirosh et al. in a recently published paper [34], compared mRNA abundance and stability between two yeast species and interestingly found a similar result by which differences in mRNA abundance were accompanied by opposite differences in stability, e.g. increase in mRNA abundance in one species was accompanied by decrease in stability in the same species. This might suggests that the negative correlation that we observe in transient responses to stress is part of a more universal feedback mechanism by which changes in stability compensate for changes in production.

Coupling of transcription and post-transcriptional regulation, and in particular by a counter action mode, is now known to be obtained by additional completely different mechanisms. One interesting mode of coupling is based on coordinated regulation by transcription factors and microRNAs which regulate shared targets [35], [36], which may allow to combine the activation of a gene with its inhibition. Thus completely different “hardware” may implement the same “software” of counter-acting regulation. Inspection of kinetics in such microRNA-transcription factor combined regulation even reveals a similar effect of the mode of coupling and the response behavior [37].

We suggest a simple molecular model which is sufficient to explain how changes in mRNA abundance are negatively coupled at the genome-wide level to change in stability in response to stress. This model is especially appealing because it shows how by a very simple manipulation, namely increasing the general imprinting probability of Rpb4/7 to transcripts, cells can achieve a genome-wide re- distribution of the general decay machinery which will result in fast relaxation of the mRNA abundance response and faster response time. Such a fast relaxation, which for induced genes, results from the coordinated increase in production and degradation, might probably lead to faster relaxation than what would be possible by increasing and then decreasing transcription, while keeping degradation constant. Our model suggests that complicated cellular responses, such as genome-wide transient mRNA changes at different temporal dynamics, can actually be controlled by simple manipulations of global cellular parameters. Of course the exact molecular details of such model remain to be validated.

## Materials and Methods

### Strains and growth conditions

All experiments were carried out using the two strains, The *rpb6* ^Q100R^ strain (*MATalpha ura3-52*, *his3delta200*, *lys2delta201*, *ade2*, *RPB6delta::HIS3* p*RPB6/CEN/LEU2*) [26] and its isogenic parental WT strain (WY37) (*(MATalpha ura3-52*, *his3delta200*, *lys2delta201*, *ade2*). Two types of experiments were conducted: experiments measuring mRNA abundance and experiments measuring mRNA decay. Both types of experiments were carried out for both strains (Figure 1 for a detailed description). For all experiments cells were grown in YPD medium (2% yeast extract, 1% peptone, 1% dextrose) at 30°C to the concentration of 1*10^7^ cells/ml. Due to a slower growth rate of the mutant, of about 40%, starters for the two strains started with slightly different concentration to ensure that both strain experiments start at the same cell concentration. To measure response to an environmental stress cells were then treated with 0.3 mM Hydrogen peroxide (H_2_O_2_) (Frutarom LTD.), or 0.1% MMS (Sigma-Aldrich) (data not shown). Survival experiments were also performed resulting in similar survival rates for both strains. For mRNA abundance measurements, aliquots (15 ml) were removed following each treatment, in the following time points: 0, 5, 10, 15, 25, 35 and 45 minutes and frozen in liquid nitrogen. RNA was extracted using MasterPure (EPICENTER Biotechnologies). The quality of the RNA was assessed using the BIOANALYZER 2100 platform (AGILENT); the samples were then processed and hybridized to Affymetrix yeast 2.0 microarrays using the Affymetrix GeneChip system according to manufacture instructions. For measuring mRNA decay a similar protocol was applied albeit with the following modifications: transcription was inhibited by adding 1,10-phenanthroline (before each experiment we prepared a fresh stock of 40 mg/ml in ethanol which was then diluted to a working concentration of 100 ug/ml) to the media 7 minutes following the addition of Hydrogen peroxide. Then aliquots were removed in the following time points: 0, 5, 10, 20, 30, 40 and 50 and were processed and hybridized as previously described. In addition we added a complete biological replicate for all mutant measurements, including half-lives in reference and oxidative stress and mRNA abundance changes in response to oxidative stress. The replicate reproduces well the presented results (Figures S2, S3, S4, S5).

### Microarray processing, determination of half-life, and mRNA abundance fold changes

All microarray samples were processed with the RMA preprocessing algorithm. In this paper unlike the work presented in our previous paper [9], transcription inhibition was achieved using the drug 1,10-phenanthroline which inhibits transcription not just from Pol II but also from Pol I, and Pol III . Thus the spike-in normalization procedure, which was used in previous decay experiments [2], [9] could not be used in this case. To get a decay profile for each gene we assumed that the global mRNA abundance decays similarly between strains and conditions and our previous measurements and scaled the data accordingly. In detail, we used as a reference the mean decay profile over the entire genome from our previous decay measurements [9]. Then each time point in the current experiment was scaled such that mean intensity over the time course would decay according to the expected reference profile. Data were then zero transformed to the first time point and exponential fit was performed on these normalized profiles. If the data was not scaled, then standard normalization procedures would result in an mRNA abundance increase for the more stable genes and a decrease for the less stable genes along the time course. It is important to note that if the slope of this change would be taken as a proxy to the genes relative stability, still the results presented in this paper will qualitatively remain the same. Also, although both of these procedures will not detect a global difference in the genome decay between the two strains, the results presented in the paper would not change because they are based on the relative rank of each gene.

Following scaling, assuming a constant decay rate throughout the course of the experiment, decay profiles were fitted to a first order exponential decay model, 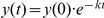, from which the fitted decay constant 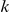 was used to calculate a gene specific half-life in each condition and strain, 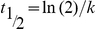. Only genes for which a high goodness of fit is achieved (R-square>0.9) were taken for further analysis. About 90% of the genes in each strain and each condition display an R-square above this cutoff indicating that this model is a good approximation to describe the decay kinetics in this data set.

For the mRNA abundance profiles, responsive genes were defined as having an absolute fold change of above 1.75 for at least one time point out of the wild type mRNA abundance measurements. These measurements were then interpolated to get a continuous response profile using a cubic spline. The maximal point was taken as the point where both the spline derivative was equal to zero and the fitted spline value reached the maximal absolute value (maximal for induced genes and minimal for repressed genes). The qualitative results presented remained robust to different response and R-square cutoffs.

For clustering the responsive mRNA abundance profiles the wild type and mutant profiles were concatenated and clustered. Distance matrix between genes was calculated using spearman rank correlation which was followed by average linkage clustering. Separation to the three clusters was done by eye using the presented dendrogram (MATLAB implementation).

All data is available at the GEO public data base under accession GSE26829.

### Microarray preprocessing for basal mRNA abundance comparisons

Standard mRNA abundance preprocessing algorithms assume a constant global distribution of mRNA levels across samples, thus any global differences between samples, e.g. an increase in the genome mean mRNA level, would not be detected. In order to compare basal mRNA abundance levels between the two strains, we normalized two zero time points, of the reference decay time courses, using spiked in RNA which followed the standard RMA normalization. We used the same normalization method described previously to normalize decay experiments[9]. Using this method mRNA levels are scaled according to an internal standard, thus a global change in the mean mRNA level would be detected. This normalization is valid here because Rpb4 and 7 are specific to Pol II which is responsible for the production of a very small amount (2–5%) of the total RNA in the cell. Thus when RNA is extracted a global difference in mRNA abundance level will be reflected in the ratio between the internal standard and the mean microarray intensity signal. As explained above, decay measurements using 1,10-phenanthroline are not suitable to such normalization because the effect is not specific to Pol II. We thus normalize all decay data sets to have the same global decay rate which results in a distribution of decay differences which is centered on zero as shown in Figure 4B. As a result, global differences between strains or conditions, as a general reduction in degradation capacity in the mutant, would be missed. Yet, the results presented in this paper will not change due to any global shifts in half-life distributions in any of the data sets.

## Supporting Information

Figure S1 Standard deviation normalized mRNA abundance profiles of cluster 1. mRNA abundance profiles from cluster 1 of Figure 3. Data is divided by the standard deviation for each gene such that the resulting profiles would have the same standard deviation. Such normalization eliminates differences in magnitude to emphasize the difference in kinetics between the two strains. Wild type mean is shown in black and the mutant in green.
(TIF)

Figure S2 Biological replicate for Figure 2B and 2C. Repetition of Figure 2B and 2C using an independent biological replicate for the mutant measurements. Data for the wild type (right panel) is taken from the first measurements.
(TIF)

Figure S3 Repetition of Figure 4A. Repetition of Figure 4A using an independent biological replicate for the mutant measurements.
(TIF)

Figure S4 Repetition of Figure 4B. Repetition of Figure 4B using an independent biological replicate for the mutant measurements.
(TIF)

Figure S5 Mutant half-life comparisons between repeats. Correlation between the half-live measurements between the two mutant biological replicates.
(TIF)

Table S1 GO enrichment analysis for clusters in Figure 3. GO categories which passed hyper geometric test with FDR (q = 0.05) for the three clusters in Figure 3.
(XLS)
