# Correction: Transcriptome Kinetics Is Governed by a Genome-Wide Coupling of mRNA Production and Degradation: A Role for RNA Pol II

**DOI:** 10.1371/annotation/7919492c-3e4b-4363-96da-f75281c1340c

**Published:** 2011-09-26

**Authors:** Ophir Shalem, Bella Groisman, Mordechai Choder, Orna Dahan, Yitzhak Pilpel

Mordechai Choder's affiliation was omitted from the published article. Dr. Choder's correct affiliation is: Department of Molecular Microbiology, Rappaport Faculty of Medicine, Technion - Israel Institute of Technology, Haifa, Israel.

