# Supplementary figures and images for: Transcriptome Kinetics Is Governed by a Genome-Wide Coupling of mRNA Production and Degradation: A Role for RNA Pol II
Source: PLoS Genet. 2011 Sep 8;7(9):e1002273. doi: 10.1371/journal.pgen.1002273 (PMC3169527; doi:10.1371/journal.pgen.1002273)

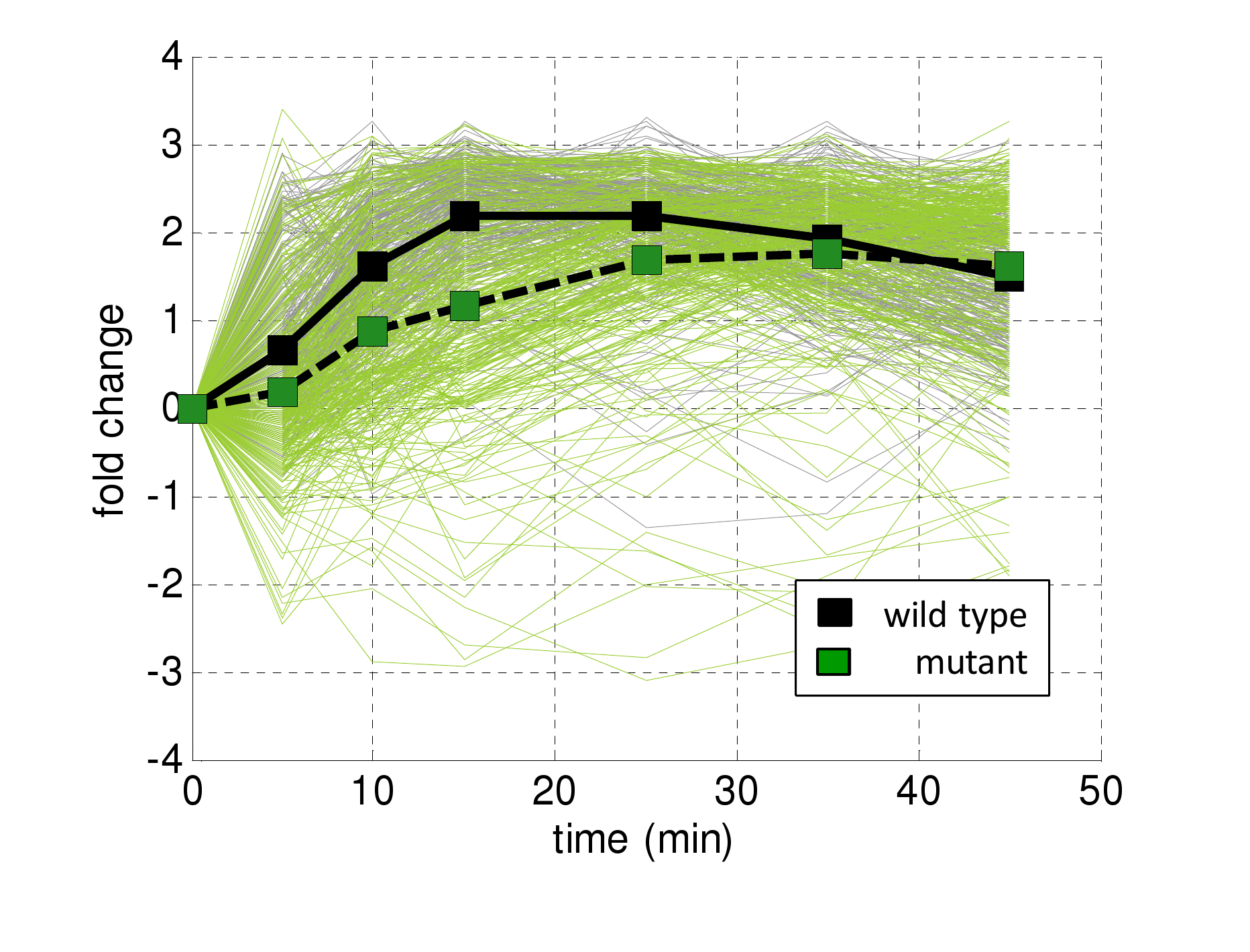

Supplement: Figure S1 — Standard deviation normalized mRNA abundance profiles of cluster 1. mRNA abundance profiles from cluster 1 of Figure 3. Data is divided by the standard deviation for each gene such that the resulting profiles would have the same standard deviation. Such normalization eliminates differences in magnitude to emphasize the difference in kinetics between the two strains. Wild type mean is shown in black and the mutant in green. (TIF) [file pgen.1002273.s001.tif]

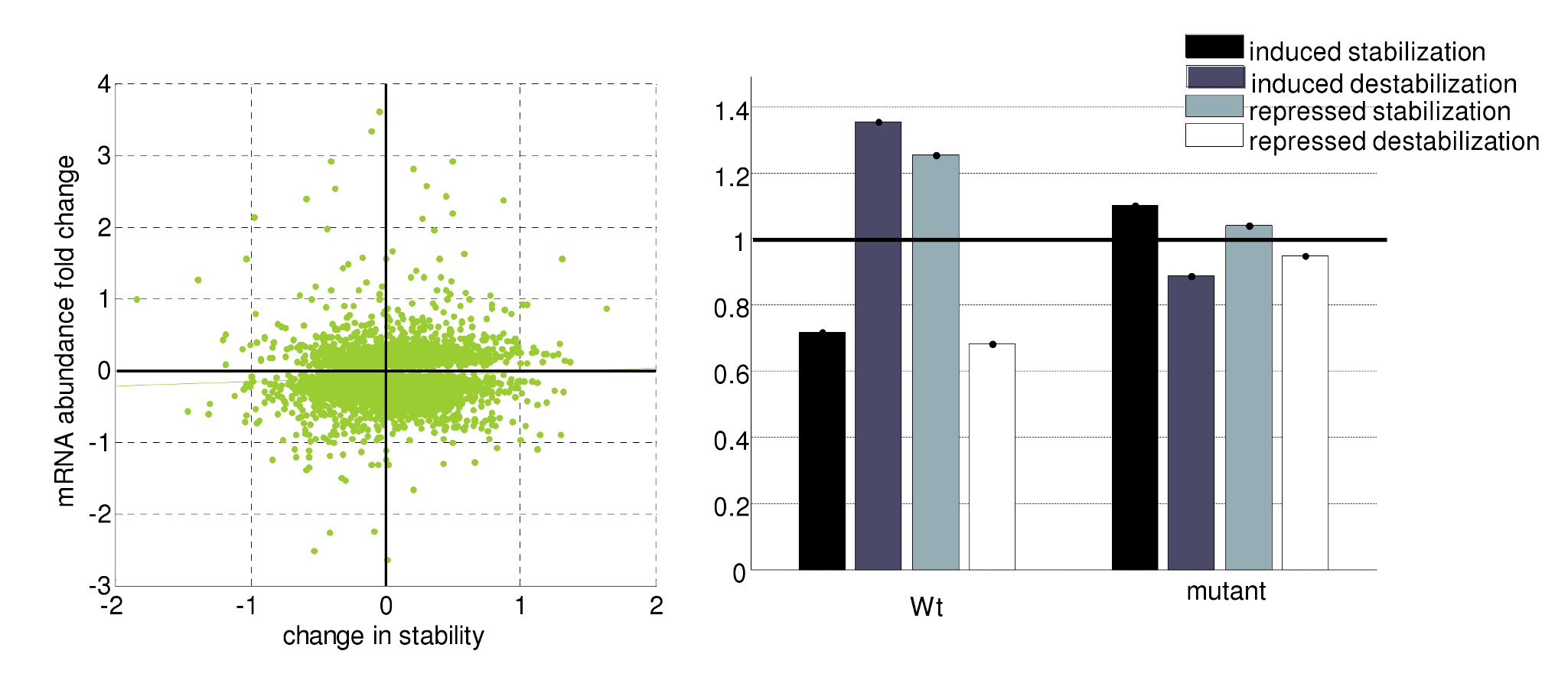

Supplement: Figure S2 — Biological replicate for Figure 2B and 2C. Repetition of Figure 2B and 2C using an independent biological replicate for the mutant measurements. Data for the wild type (right panel) is taken from the first measurements. (TIF) [file pgen.1002273.s002.tif]

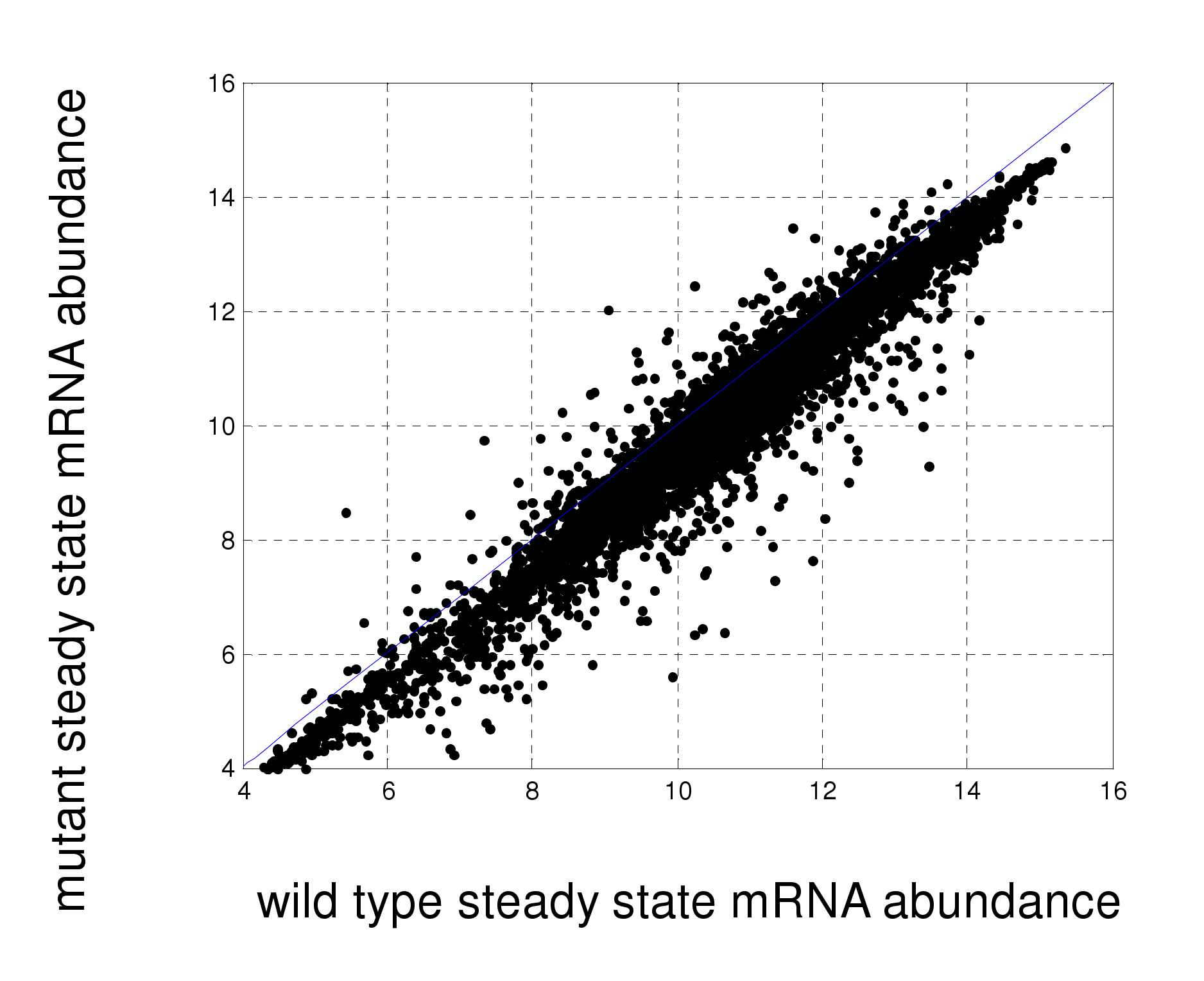

Supplement: Figure S3 — Repetition of Figure 4A. Repetition of Figure 4A using an independent biological replicate for the mutant measurements. (TIF) [file pgen.1002273.s003.tif]

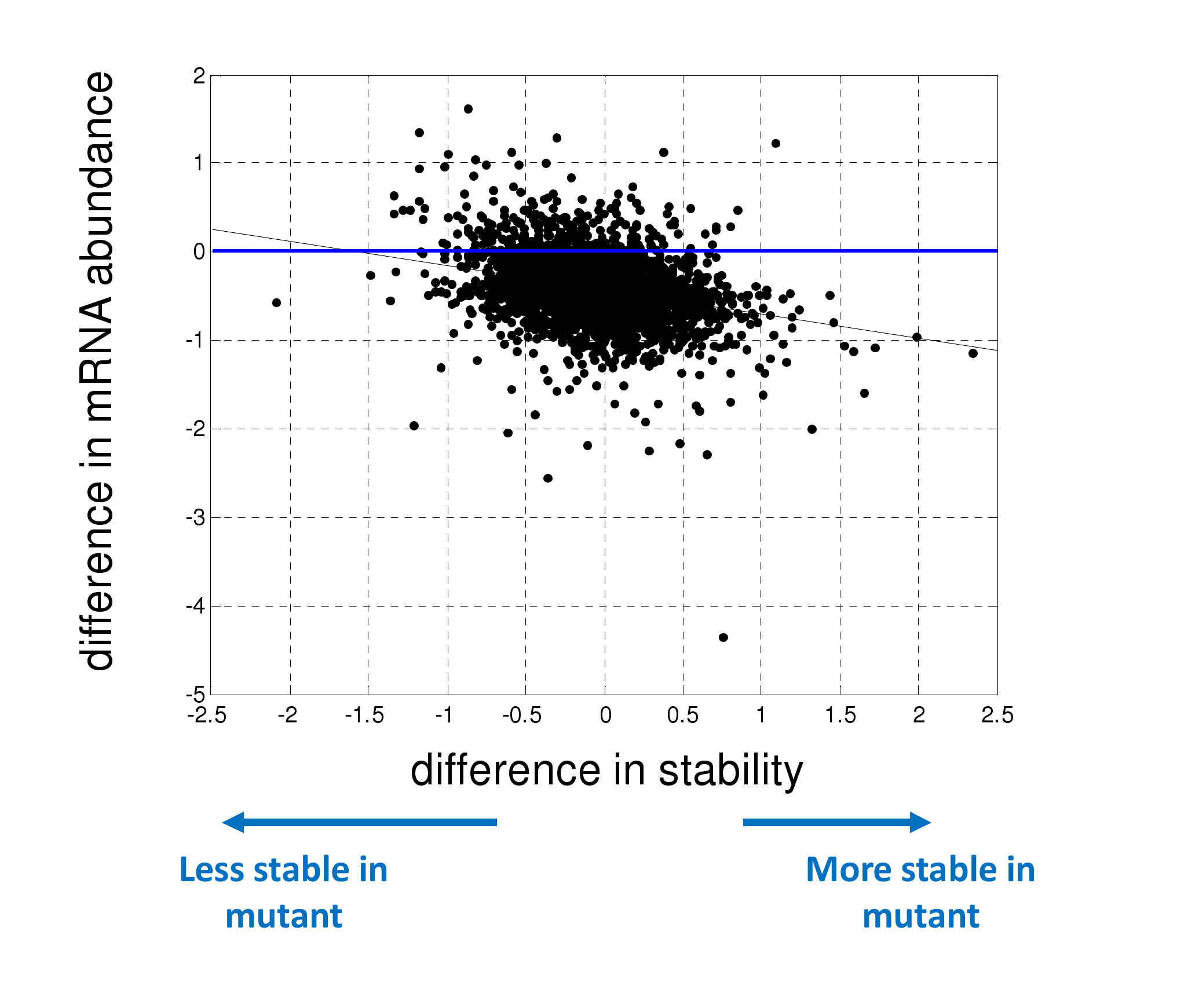

Supplement: Figure S4 — Repetition of Figure 4B. Repetition of Figure 4B using an independent biological replicate for the mutant measurements. (TIF) [file pgen.1002273.s004.tif]

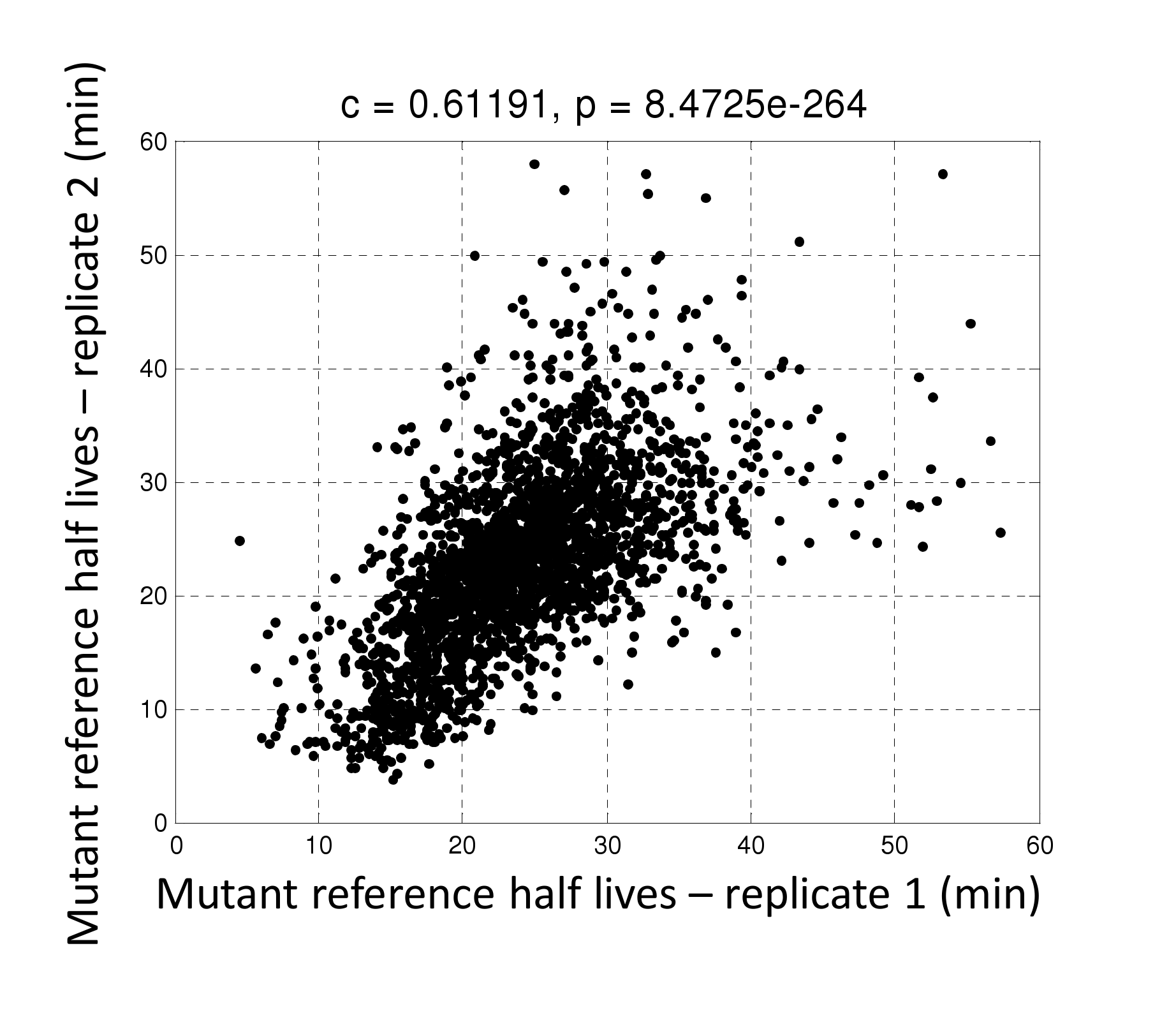

Supplement: Figure S5 — Mutant half-life comparisons between repeats. Correlation between the half-live measurements between the two mutant biological replicates. (TIF) [file pgen.1002273.s005.tif]
